# Dark Rearing Promotes the Recovery of Visual Cortical Responses but Not the Morphology of Geniculocortical Axons in Amblyopic Cat

**DOI:** 10.3389/fncir.2021.637638

**Published:** 2021-04-16

**Authors:** Takahiro Gotou, Katsuro Kameyama, Ayane Kobayashi, Kayoko Okamura, Takahiko Ando, Keiko Terata, Chihiro Yamada, Hiroyuki Ohta, Ayaka Morizane, Yoshio Hata

**Affiliations:** ^1^Division of Integrative Bioscience, Tottori University Graduate School of Medical Sciences, Yonago, Japan; ^2^Division of Neuroscience, School of Life Science, Faculty of Medicine, Tottori University, Yonago, Japan

**Keywords:** visual cortex, geniculocortical axons, dark rearing, ocular dominance, monocular deprivation, amblyopia

## Abstract

Monocular deprivation (MD) of vision during early postnatal life induces amblyopia, and most neurons in the primary visual cortex lose their responses to the closed eye. Anatomically, the somata of neurons in the closed-eye recipient layer of the lateral geniculate nucleus (LGN) shrink and their axons projecting to the visual cortex retract. Although it has been difficult to restore visual acuity after maturation, recent studies in rodents and cats showed that a period of exposure to complete darkness could promote recovery from amblyopia induced by prior MD. However, in cats, which have an organization of central visual pathways similar to humans, the effect of dark rearing only improves monocular vision and does not restore binocular depth perception. To determine whether dark rearing can completely restore the visual pathway, we examined its effect on the three major concomitants of MD in individual visual neurons, eye preference of visual cortical neurons and soma size and axon morphology of LGN neurons. Dark rearing improved the recovery of visual cortical responses to the closed eye compared with the recovery under binocular conditions. However, geniculocortical axons serving the closed eye remained retracted after dark rearing, whereas reopening the closed eye restored the soma size of LGN neurons. These results indicate that dark rearing incompletely restores the visual pathway, and thus exerts a limited restorative effect on visual function.

## Introduction

Experimental induction of amblyopia is a good model of the experience-dependent development of brain function and remodeling of the neural circuit. Monocular deprivation (MD) of vision in cats during early postnatal life impairs the visual acuity of the closed eye (Giffin and Mitchell, 1978). As a neural correlate, the neurons in the primary visual cortex reduce their responses to the closed eye as revealed by recording single-unit spike activity (Wiesel and Hubel, 1963b) and visually evoked potentials (Snyder and Shapley, 1979). Anatomically, MD shrinks neuronal somata in the closed-eye recipient layer of the lateral geniculate nucleus (LGN) that mediates retinal inputs to the visual cortex (Wiesel and Hubel, 1963a). Besides, the geniculocortical axons carrying information from the closed eye show a retraction of cortical arbors (Antonini and Stryker, 1996) and shrinkage of their cortical territory (Shatz and Stryker, 1978). These changes, referred to as ocular dominance (OD) plasticity, are observed only during the critical period of early postnatal life and are difficult to induce in adults (Hubel and Wiesel, 1970; Olson and Freeman, 1980).

Restoration of vision to the closed eye can reverse the effect of MD if the deprivation is brief. Simply reopening the closed eye during the critical period induces substantial recovery of visual acuity in cats. The restored acuity can be comparable to the open eye (Mitchell et al., 1977; Mitchell, 1988) or still somewhat impaired (Duffy and Mitchell, 2013; Murphy et al., 2015). Cortical responses to the closed eye also restore to a level comparable to the open eye, although the number of neurons with binocular responses remains small (Mitchell et al., 1977; Olson and Freeman, 1978). Recovery from MD becomes more difficult after prolonged deprivation beyond the critical period (Wiesel and Hubel, 1965; Mitchell et al., 1977; Mitchell, 1988).

Recent studies have reactivated OD plasticity in adult rodents through various interventions. Molecular interventions such as knockouts of a myelin-associated factor (Nogo-66 receptor) or of a modulator of the nicotinic acetylcholine receptor (lynx1) reactivate OD plasticity in the visual cortex of adult mice (McGee et al., 2005; Morishita et al., 2010). Pharmacological interventions such as the administration of chondroitinase-ABC to degrade chondroitin sulfate proteoglycans in the visual cortex, chronic administration of the antidepressant fluoxetine, or administration of a subanesthetic dose of ketamine also restore OD plasticity in adult rodents (Pizzorusso et al., 2002; Maya-Vetencourt et al., 2008; Grieco et al., 2020). Additionally, noninvasive rearing conditions such as environmental enrichment or exposure to total darkness induce OD plasticity in adult rodents (He et al., 2006; Sale et al., 2007). While these rodent studies show that recovery from amblyopia is possible, the effectiveness of these interventions in higher mammals such as humans, monkeys, and cats require confirmation.

Dark rearing, a noninvasive intervention, improves the recovery of visual acuity of the amblyopic eye in behavioral experiments on cats (Duffy and Mitchell, 2013) while it often fails to restore binocular depth perception (Mitchell et al., 2016). It is still unclear which part of the early visual pathway dark rearing enhances recovery. To clarify this point, we determined whether dark rearing can enhance the restoration from the three major effects of MD on individual neurons in the early visual pathway, eye preference of visual cortical neurons, soma size of LGN neurons, and morphology of geniculocortical axons in cats. We found that dark rearing improves the recovery of visual cortical responses to the amblyopic eye but does not restore the morphology of geniculocortical axons, whereas simply reopening the closed eye restores the soma size of LGN neurons.

## Materials and Methods

### Animals

All cats in this study were born in the breeding colony of the Tottori University Research Center for Bioscience and Technology. The animal care and use committee of the Tottori University approved all experimental procedures (approval number: 14-Y-18).

### Visual Manipulation

**Figure 1:**
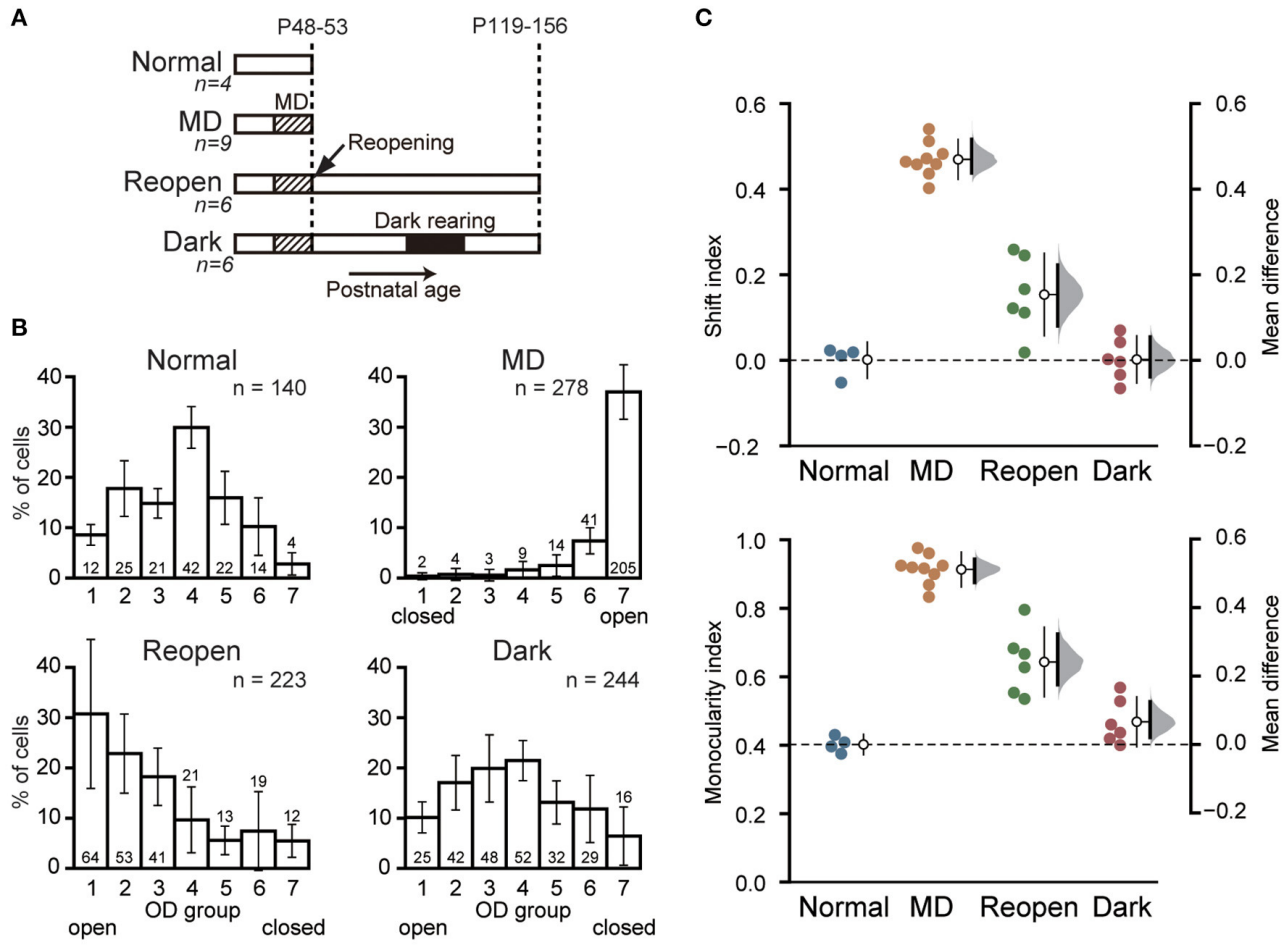
Dark rearing promotes the recovery of cortical responses to the closed eye. **(A)** Schematic illustration showing the experimental procedures and schedule of the four animal groups. Normal: Normal young animals. Electrophysiological recording was made at P48–53. MD: Young animals with monocular deprivation (MD). Recording was made after 6–7 days of MD. Reopen: Control animals that were raised in binocular conditions after MD. Recording was made at adolescence. Dark: Animals that were raised in binocular conditions after MD and reared in darkness for 10 days at adolescence. Recording was made 12–20 days after dark rearing. The number of animals is given below each group name. **(B)** Ocular dominance (OD) of the primary visual cortical neurons in each animal group. The closed eye is indicated below the OD score. Each histogram shows the mean and SD of all animals in each group. The number of neurons in each OD score is described at each column. The total number of neurons is indicated at top-right of each histogram. Data of Normal and MD groups were obtained in our previous study (Morishima et al., 2013). **(C)** Comparison of OD distribution among animal groups. Each symbol represents the shift index (top) and the monocularity index (bottom) of individual animals. The open circles with thin vertical bars indicate the mean and SD of each group. The thick vertical bars represent the 95% confidence interval of mean difference with a bootstrap sampling distribution plotted on the right axes.

At postnatal days (P) 41–46, kittens were deprived of vision in one eye (MD) (Figure 1A). Animals were anesthetized with isoflurane (Isoflurane, Pfizer, Japan) (2–5% in O_2_). Their eyelids were sutured shut and surgical adhesives (Aron Alpha A, Sankyo, Japan) were applied to the junction. The eyelids were opened after 6–7 days of MD and the animals were reared in binocular conditions thereafter. The animals were separated into the test group (Dark group) and the control group (Reopen group). Animals in the Dark group were kept in complete darkness for 10 days at around P100. During dark rearing, we took care of them and checked their condition every day using infrared googles. After dark rearing, the animals were placed in conventional cages for 12–20 days before the physiological experiments. Animals in the Reopen group were kept in conventional cages after the eyelids were opened until the physiological experiments. Rearing conditions of all animals in the present study are summarized in Table 1 with the information of data obtained.

**Table 1:** Animal list with rearing conditions and obtained data.

		**MD**		**Dark Age**	**Recording (perfusion)**				
**Condition**	**Animal ID**	**Age**	**Eye**		**Age**	**Hem**.	**# units**	**# arbors**	**LGN**
Normal	10-08d				50	R	37*		L
	10-08e				50	R	32*		L
	11-01b				49	R	40*		L
	11-03a				49	R	31*		
	19-04b				49				L, R
MD	05-07B	43	L		50	R	29*		
	06-05E	46	R		53	L	34*		
	06-13C	42	L		48	R	31*		R
	07-06b	41	L		47	R	28*		R
	07-11d	42	L		48				R
	07-11e	43	L		49				R
	07-12d	45	L		51	R	32*		
	07-13c	42	L		48				R
	07-17d	41	L		50	R	33*		
	07-18b	41	L		47	R	30*		
	07-19e	45	L		51	R	30*		
	08-02c	45	L		51	R	31*		
	19-07c	42	R		48				L, R
Reopen	11-09b	44	L		156	L	31		L, R
	12-01a	43	L		151	L	33	4(C)	
	14-07a	42	L		119	L	41		
	14-07b	42	L		121	L	50		
	16-01a	42	R		123	R	34		L, R
	16-01b	42	R		126	R	34	3(C)	L, R
Dark	13-04d	42	R	98	122	L	42	3(C), 1(O)	L, R
	13-04e	42	L	98	124	L	41	1(C), 1(O)	
	14-01d	42	L	98	120	L	44	2(C), 2(O)	L, R
	14-01e	42	L	98	122	L	42		L, R
	16-02a	42	R	98	128	R	40	1(C), 3(O)	L, R
	16-02b	42	R	98	120	R	35	3(C), 6(O)	L, R

*The first number of the ID indicates the year of birth. The age of starting MD, dark rearing, and unit recording are shown as postnatal days, with total number of units recorded in each animal. Asterisks denote the data quoted from our previous study (Morishima et al., 2013). C and O in the “# arbors” column indicate the arbor serving the closed eye and open eye, respectively. The “LGN” column indicates the hemisphere of LGN used for soma size measurement*.

### Tracer Injection

Neurons at layer A of the LGN were labeled 10–13 days before perfusion using biotinylated dextran amine (BDA) (10% in 0.5 M NaCl, BDA-10,000, Molecular Probes, OR, USA). Animals were sedated with medetomidine (0.05 mg/kg i.m., Dorbene, Kyoritsu Seiyaku, Japan) 10 min before anesthesia with isoflurane (1.5–3.5% in O_2_). Animals were retained in a stereotaxic instrument. A glass pipette filled with the BDA solution was inserted stereotaxically to locate a clear visual response from layer A of the LGN in each hemisphere. BDA was iontophoretically injected (pipette positive current of 4 μA, 2 s on/off pulse, 75 times) at 1–2 sites in each LGN. All surgical procedures were performed under sterile condition. Body temperature, heart rate, respiration rate, and end-tidal CO_2_ concentration were continuously monitored throughout the surgery. All the incisions were infiltrated with local anesthetics (Xylocaine pump spray, AstraZeneca, Japan). The animals were given an antimicrobial agent (enrofloxacin, 5 mg/kg s.c., Baytril, Bayer, Germany) every day after the surgery.

### Electrophysiology

Single-unit activity was recorded from the primary visual cortex to evaluate the OD of individual cortical neurons. Animals were sedated with medetomidine, given buprenorphine hydrochloride (0.02 mg/kg i.m., Lepetan, Otsuka, Japan) for analgesia, and anesthetized with isoflurane (1.5–3.5% in O_2_). Their pupils were dilated with 0.5% tropicamide and 0.5% phenylephrine hydrochloride (Mydorin-P, Santen, Japan), and contact lenses were placed on the corneas. The animals were paralyzed with gallamine triethiodide (10 mg/kg/h, i.v., Sigma-Aldrich, MO, USA) and maintained under artificial respiration. The end-tidal CO_2_ concentration and body temperature were maintained at 4% and 38°C, respectively. All of the incisions were infiltrated with local anesthetics.

Single-unit activity was recorded with a tungsten electrode (10 MΩ, FHC, ME, USA). Neural activity was filtered at 500–5,000 Hz and amplified 1,000-fold by an amplifier (Model 1800, A-M systems Inc., WA, USA). The visual response of cortical neurons was induced by orientated light stimulations to each eye, and the OD of individual neurons was determined based on a conventional seven-point classification by comparing the strength of the response to each eye aurally (1–7: a score of 1 corresponds to the contralateral eye alone, a score of 7 corresponds to the ipsilateral eye alone, and a score of 4 is for equal binocular responses) (Hubel and Wiesel, 1962). The OD distribution in each hemisphere was quantified by calculating the contralateral bias index (CBI) as follows:

$$\begin{array}{l} \text{CBI}=\left[\left(\text{n}1-\text{n}7\right)+2/3\;\left(\text{n}2-\text{n}6\right)+1/3\;\left(\text{n}3-\text{n}5\right)+N\right]/2N \end{array}$$

where *ni* is the number of cells in OD group *i* and *N* is the total number of visually responsive cells. We then calculated the shift index (SI) to evaluate the deviation of the OD distribution from that of the normal animals as follows:

$$\begin{array}{l} \text{SI}=\text{CBI}-\text{Average CBI of normal animals}. \end{array}$$

We also evaluated the monocular tendency of responsiveness as follows:

$$\begin{array}{l} \text{Monocularity index }\left(\text{MI}\right)\\ \;=\left[\left(\text{n}1+\text{n}7\right)+2/3\;\left(\text{n}2+\text{n}6\right)+1/3\;\left(\text{n}3+\text{n}5\right)\right]/N. \end{array}$$

### Histology

After recording, the animals were euthanized with an overdose of pentobarbital sodium (150 mg/kg i.v., Somnopentyl, Kyoritsu, Japan) and perfused transcardially with a cold Ringer's solution and 4% paraformaldehyde in 0.1 M phosphate buffer (PB, pH 7.4). Their brain was removed and postfixed overnight. Tissue blocks containing the LGN and visual cortex were cut at the frontal plane using a freezing microtome (thickness of the visual cortex and LGN, 60 and 80 μm, respectively). The sections were washed in phosphate-buffered saline (PBS) and incubated in a blocking solution composed of 5% bovine serum albumin (BSA, Sigma-Aldrich, MO, USA), 3% normal rabbit serum (Vector Laboratories, CA, USA), and 0.7% Triton X-100 (Wako Chemicals, Japan) in PBS. The sections were then washed with a wash buffer (0.1% BSA and 0.1% Triton X-100 in PBS), transferred to a solution of avidin-biotin-HRP complex (Vector Laboratories, CA, USA), and maintained at 4°C overnight. After washing the sections with the wash buffer, they were reacted with a biotinylated tyramine-glucose oxidase solution for tyramide signal amplification (Kuramoto et al., 2009). The sections were then processed using the standard ABC kit (Vector Laboratories, CA, USA) and reacted with 0.1% diaminobenzidine in hydrochloride, 1% cobalt chloride, 1% nickel ammonium sulfate, 0.00025% hydrogen peroxide to visualize the tracer. All sections were dehydrated in a graded series of ethanol and defatted in xylene overnight. Selected sections containing the LGN were stained with cresyl violet to localize the layer boundaries and injection sites, and to measure the soma size of the LGN neurons. Sections were coverslipped with DPX mountant (Sigma-Aldrich, MO, USA).

### Measurement of LGN Soma Size

For each animal, the coronal sections of the LGN at the middle of the anterior-posterior axis, which correspond to the horizontal meridian of the visual field (Sanderson, 1971), were selected for soma size measurement. Regions-of-interest (ROIs) of 243 μm × 183 μm were set at six sites in the medial half of layers A and A1 of the LGN. Images of each ROI were captured using a microscope (BZ-9000, Keyence, Japan) with a × 60 microscope objective through the full thickness of each section at a 2 μm step. Cells with dark cytoplasmic and nucleolar staining and pale nuclear staining were considered as neurons (Duffy et al., 2014). They were all sampled for area measurement in each ROI (228–457 cells in each layer). Neuron size was measured by delineating soma using the image processing software Fiji (Schindelin et al., 2012) at the depth in which cell nucleoli were clearly observed, thus providing the cell's maximum diameter at the presumed soma midline. Soma size measurement was performed in blind condition. The images of the specimen were given to the measurers without the information about the rearing condition or LGN layer.

The areas of all measured soma in individual ROIs were averaged and the average of all ROIs in each layer of individual animals was used as the representative value of the layer. The difference in soma size in layers A and A1 of individual animals was evaluated by calculating the shrinkage index as follows:

$$\begin{array}{l} \text{Shrinkage index}=\left(\text{A}-\text{A}1\right)/\left(\text{A}+\text{A}1\right)\\ -\left({\text{A}}_{\text{norm}}-\text{A}1_{\text{norm}}\right)/\left({\text{A}}_{\text{norm}}+\text{A}1_{\text{norm}}\right) \end{array}$$

where A and A1 are the representative values of soma size in each layer and the “norm” suffix indicates the value for the normal animals. The index takes a negative value when neurons in layer A of each animal show a shrinkage. When layer A1 was the recipient of the closed eye, the sign of the index was inversed. Thus, the shrinkage index represents the degree of soma size shrinkage in the closed-eye layer compared with the normal animals.

### Axonal Arbor Analysis

We first determined whether the injection sites of BDA were well confined to layer A of the LGN. When an injection site invaded layer A1 (corresponding to the ipsilateral eye), we eliminated all samples from the same hemisphere from the arbor reconstruction. We reconstructed the cortical arbors of the individual BDA-filled axons from serial sections in three dimensions with the aid of computer software (Neurolucida, Microbrightfield, VT, USA). We calculated two measures, the branch point number and the total length, to quantify the complexity and size of individual axonal arbors using original programs (MATLAB, MathWorks, MA, USA). The branch point number is the number of axon bifurcations in the terminal arborization. We obtained the total length by adding the lengths of all axonal segments constituting the terminal arborization together. For the analysis, we used only the portion of the arbor located in layers II/III and IV.

### Statistics

Comparison among experimental groups was performed using estimation statistics. The 95% confidence intervals of the mean difference were calculated using bootstrap resampling (5,000 resampling with replacement) with DABEST software (https://www.estimationstats.com/) (Ho et al., 2019). Significance testing was performed using the EZR software (Jichi Medical University, Japan) (Kanda, 2013), which is a graphical user interface for R (version 2.13.0, The R Foundation for Statistical Computing, Austria). We examined the normality of the sample distribution of all data using the Shapiro–Wilk test. Statistical comparisons between two groups were conducted using an unpaired *t*-test or the Mann–Whitney *U*-test. Multiple comparisons were conducted using the Tukey–Kramer test or Steel–Dwass test. We used the Fisher's exact test for comparison of distribution.

## Results

### Dark Rearing Restores Binocular Response of Cortical Neurons in Amblyopic Cats

To determine whether dark rearing restores visual cortical responses to amblyopic eye, we prepared amblyopic animals. We deprived them of vision in one eye at a young age by suturing their eyelid for 6–7 days (Figure 1A, Reopen and Dark). We then opened the closed eye and raised them under binocular conditions. We later reared the adolescent animals of the Dark group in complete darkness for 10 days followed by 2 weeks under normal conditions. We compared OD of neurons in the primary visual cortex between these two groups (Reopen and Dark) and with the normal and MD animals (Figure 1A, Normal and MD) obtained in our previous study (Morishima et al., 2013) (Table 1).

Figure 1B represents the OD histograms obtained in the four groups. Most neurons in the normal animals showed binocular responses and the OD histogram had a peak at OD score 4 with a slight preference for the contralateral eye (Figure 1B, Normal). MD at young age drastically reduced cortical responses to the closed eye and most neurons responded only to the open eye (Figure 1B, MD). In the Reopen group, the visual cortical neurons restored binocular responsiveness to some extent but still showed a clear preference for the eye that was not sutured shut (Figure 1B, Reopen). In the Dark group, neurons recovered more responsiveness to the closed eye, and the histogram was similar to that of the normal animals (Figure 1B, Dark). To examine the differences in the histograms in more detail, we compared the histograms of the Reopen and Dark groups with that of the Normal group for each of the OD scores (Supplementary Figure 1). The Reopen group had a higher proportion of cells that respond only to the open eye (OD1) and less proportion of strongly binocular cells (OD4 and 5) (OD1: mean difference = 22.168% [95% confidence interval (CI): 8.948, 31.377], OD4: −20.261 [95%CI: −24.494, −14.733], OD5: −10.342 [95%CI: −14.132, −3.779]). On the other hand, the Dark group showed a small reduction in the proportion of strongly binocular cells (OD4) (−8.462 [95%CI: −13.212, −4.007]. The extent of reduction of OD4 cells was more significant in the Reopen group than the Dark group (mean difference between Reopen and Dark = 11.799 [95%CI: 6.411, 17.682]).

To compare the OD distribution among animal groups quantitatively, we calculated the SI, which indicates the deviation of eye preference from that of the normal animals, and the MI, which indicates the degree of monocular responsiveness in each animal (Figure 1C). As expected from OD histograms, the SI and MI of the MD group (SI = 0.489 ± 0.040, MI = 0.914 ± 0.043) were significantly different from those of the Normal group (SI = 0.000 ± 0.035, mean difference = 0.469 [95%CI: 0.437, 0.517], MI = 0.402 ± 0.023, 0.511 [95%CI: 0.474, 0.541], SI: *p* < 0.0001, MI: *p* < 0.0001, Tukey–Kramer test). The SI and MI of the Reopen group (SI = 0.153 ± 0.090, MI = 0.644 ± 0.095) were smaller than those of the MD group but still larger than those of the Normal group (SI: 0.153 [95%CI: 0.080, 0.223], *p* = 0.0025, MI: 0.241 [95%CI: 0.175, 0.323], *p* < 0.0001) indicating a partial recovery of cortical responses to the closed eye. On the other hand, we found no difference between the Dark and Normal groups in SI (0.002 ± 0.049, 0.002 [95%CI: −0.039, 0.055], *p* = 0.99995) and a very small increase in MI (0.468 ± 0.066, 0.066 [95%CI: 0.021, 0.125], *p* = 0.386). In conclusion, binocular rearing by reopening the closed eye induced a restoration of cortical responses to the closed eye and dark rearing significantly improved the recovery.

### Dark Rearing Does Not Restore the Morphology of Geniculocortical Axons

**Figure 2:**
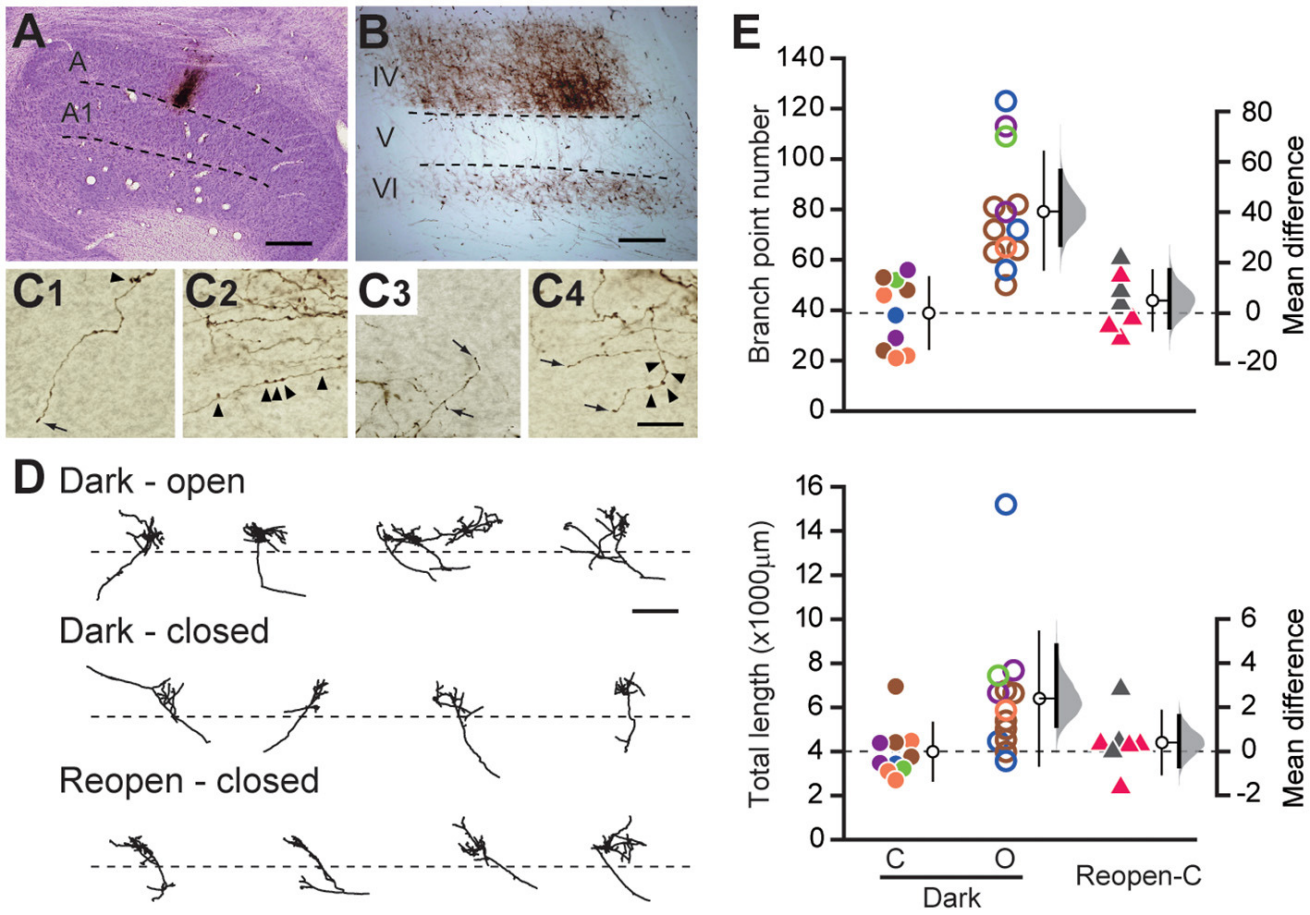
Geniculocortical axons serving the closed eye remain retracted after dark rearing. **(A)** Example of a Nissl-stained coronal section of the lateral geniculate nucleus containing a BDA injection site in layer A. Scale: 500 μm. **(B)** Low magnification view of the labeled geniculocortical axons in the primary visual cortex. Dense arborization is observed in layer IV. Scale: 200 μm. **(C1–C4)** High magnification view of the labeled axons in the visual cortex. The arrows and arrowheads indicate the terminal swellings and varicosities of axons, respectively. Scale: 20 μm. **(D)** Drawings of reconstructed axons serving the open eye (top) and the closed eye (middle) of the Dark group, and those serving the closed eye in the Reopen group (bottom). The dotted lines indicate the border between layer IV and V. Scale: 500 μm. **(E)** Comparison of axonal arbor morphology among animal groups. Each symbol represents the number of branch points (top) and the total length (bottom) of individual axons. The symbols with the same color represent data obtained from the same animal. The other conventions are the same as in Figure 1C. O, open-eye axon; C, closed-eye axon.

MD during the critical period induces not only functional changes in the visual cortical neurons but also a retraction of geniculocortical axons serving the closed eye (Antonini and Stryker, 1996). Therefore, we next examined whether dark rearing has a restorative effect on axon morphology. To visualize the afferent axons from the LGN, we injected an anterograde tracer (BDA) into layer A of the LGN, which receives inputs from the contralateral eye (Figure 2A). We observed the labeled afferent axons mainly in layer IV of the visual cortex (Figure 2B). At high magnification, individual axons were labeled well so that fine structures such as axon terminals and varicosities could be clearly observed (Figure 2C). We traced and reconstructed the cortical arborization of individual axons in three dimensions as exemplified in Figure 2D.

We compared the complexity and size of the cortical arbors of the axons serving the closed eye and the open eye in the Dark group by calculating the number of branch points and the total length of the terminal arborization (Figure 2E). Even after dark rearing, the closed eye axons (branch point: 38.9 ± 13.8, length: 3,995.0 ± 1,201.7 μm, *n* = 10) were significantly simpler and shorter than those of the open eye (branch point: 79.2 ± 22.6, mean difference = 40.254 [95%CI: 27.046, 56.500], *p* = 0.0004, length: 6,403.6 ± 2,955.1 μm, 2408.598 [95%CI: 1,166.122, 4,819.545], *p* = 0.0099, *n* = 13, Steel–Dwass test). Besides, we found no difference in the morphology of closed eye axons between the Dark group and the Reopen group (Reopen closed-eye axon, branch point: 43.7 ± 11.4, 4.814 [95%CI: −5.942, 16.886], *p* = 0.85, length: 4,408.4 ± 1,307.5 μm, 413.361 [95%CI: −669.126, 1,613.640], *p* = 0.922, *n* = 7). Therefore, dark rearing did not restore the morphology of the retracted axons.

### Reopening the Closed Eye Restores LGN Soma Size

**Figure 3:**
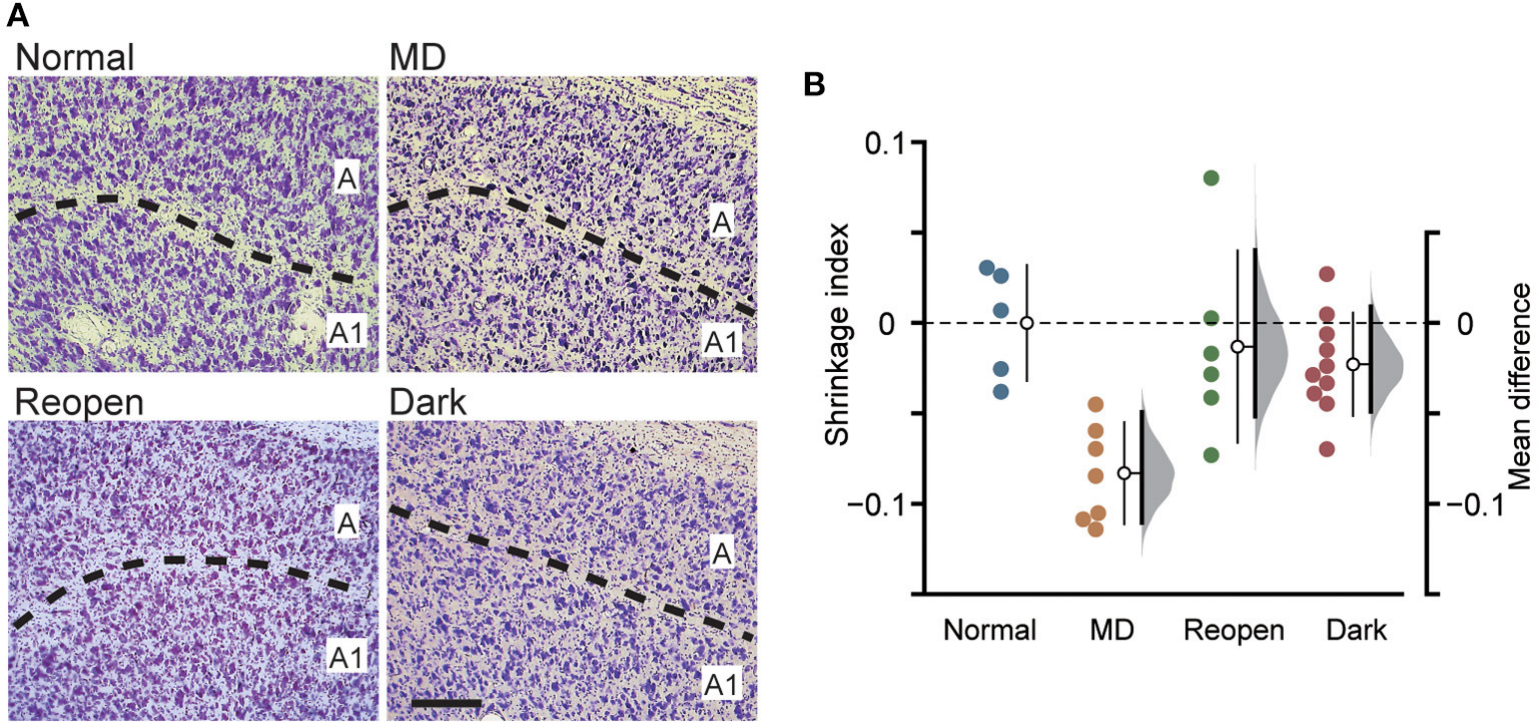
Measurement of the soma size of LGN neurons. **(A)** Examples of the Nissl-stained sections of the LGN in each animal group. The dotted lines represent the border between layer A and A1. Scale: 200 μm. **(B)** Each symbol represents the shrinkage index of individual animals. The other conventions are the same as in Figure 1C.

MD induces a remarkable shrinkage of neuronal somata in the closed-eye recipient layer of the LGN (Wiesel and Hubel, 1963a). While dark rearing did not restore the cortical arbor morphology of LGN neurons, binocular vision (O'Leary et al., 2012) and binocular visual deprivation such as dark rearing and binocular eyelid suture (Duffy et al., 2015) restored the soma size of shrunk LGN neurons. To examine the restoration of LGN soma size in the present protocol, we measured the cross-sectional area of the soma of LGN cells in coronal sections of each animal group (Figure 3A; Supplementary Figure 2) and evaluated the degree of shrinkage by calculating the shrinkage index (Figure 3B). The Normal group had a slightly but significantly larger soma area in layer A1 than in layer A (layer A: 157.141 ± 45.689 μm^2^, A1: 171.320 ± 42.469 μm^2^, mean difference = 14.180 [95%CI: 7.859, 21.118], *p* < 0.05, paired *t*-test in five animals; Supplementary Figure 2, Normal), which is consistent with previous reports (Hickey et al., 1977; Bear and Colman, 1990). Soma area was smaller in the closed eye recipient layer in the MD group, but the tendency appears weak in the Reopen and Dark groups (Supplementary Figure 2). To compare the soma area among animal groups correcting for innate difference between layers, we evaluated the degree of shrinkage by calculating the shrinkage index (Figure 3B). Compared to the Normal group, MD significantly shrunk soma size in the closed-eye layer (shrinkage index, Normal: 0.000 ± 0.031, *n* = 5, MD: −0.083 ± 0.027, *n* = 7, mean difference = −0.084 [95%CI: −0.111, −0.050], *p* < 0.005, Tukey–Kramer test). In the Reopen and Dark groups, we did not find shrinkage compared to the Normal group (Reopen: −0.023 ± 0.027, *n* = 6, −0.013 [95%CI: −0.051, 0.041], *p* = 0.635, Dark: −0.014 ± 0.054, *n* = 10, −0.023 [95%CI: −0.049, 0.009], *p* = 0.907). Also, we found no shrinkage difference between the Reopen and Dark groups (mean difference = 0.010 [95%CI: −0.023, 0.064], *p* = 0.962). Therefore, in the present experiments, the binocular recovery period following MD successfully restored the soma size in the closed-eye layer of the LGN.

## Discussion

In the present experiments, dark rearing for 10 days at adolescence improved the recovery of binocular responsiveness of cortical neurons to a level comparable to those of normal animals. Alternatively, dark rearing did not restore the morphology of geniculocortical axons.

Previous studies using behavioral test (Mitchell, 1988; Murphy et al., 2015) and single unit recording (Mitchell et al., 1977; Olson and Freeman, 1978) demonstrated that visual acuity of and cortical neurons' responses to the closed eye can restore when binocular vision was given after brief MD. Consistent with these results, we found considerable recovery of cortical responses to the closed eye in the Reopen group. Therefore, simply reintroducing vision to the closed eye can restore the cortical response. However, recovery by binocular vision is not a complete recovery of OD distribution. The previous studies showed a significant reduction of binocularly responsive neurons compared to normal animals. On the other hand, in the present study, the OD histogram of the Reopen group remained shifted toward the open eye. This difference might be explained by a difference in age of MD. Olson and Freeman (1978) gave MD during P28-38 and found 37% of neurons dominated by the closed eye after recovery. We started binocular recovery at P48-50 after 6-day MD and found 20% of neurons dominated by the closed eye. Mitchell et al. (1977) gave MD until P45 and 60 and found 31 and 19% of neurons dominated by the closed eye, respectively. The later binocular recovery starts, the smaller number of neurons are dominated by the closed eye. Therefore, the timing of reopening appears important to the effectiveness of binocular recovery. The present results confirm the restorative effect of binocular vision after MD and further demonstrated that dark rearing can enhance the recovery.

Most previous studies evaluated the effect of dark rearing on the recovery of visual cortical responses using visual evoked potentials. The visual evoked potential reflects a complex of transmembrane currents composed mainly of synaptic currents along with other sources such as action potentials and various ionic currents (Buzsáki et al., 2012). Whereas many studies had demonstrated the recovery from monocular deprivation by measuring spike activity (Wiesel and Hubel, 1965; Blakemore and Van Sluyters, 1974; Mitchell et al., 1977; Olson and Freeman, 1978), which is more reflective of neuronal output, few studies have used single-unit recording to explore the effects of dark rearing. In rats, dark rearing followed by reversing the eye deprivation restored spike responses of individual cortical neurons to the originally closed eye (Montey and Quinlan, 2011). As for the recovery under normal binocular conditions, a previous study using two-photon Ca^2+^ imaging of individual neurons failed to find significant recovery of cortical responses to the closed eye following dark rearing (Erchova et al., 2017). It might have been difficult to find a significant effect of dark rearing because, in mice, considerable cortical plasticity remains after the critical period (Sawtell et al., 2003), thus reopening the closed eye and raising under binocular conditions might have led to a complete recovery of binocular responses. The present results confirm that dark rearing promotes the recovery of spike responses to the closed eye in cats and suggest a neural substrate of behavioral restoration of the amblyopic eye reported previously (Duffy and Mitchell, 2013).

While dark rearing improved the recovery of visual cortical responses to the closed eye, geniculocortical axons serving the closed eye were still shorter and had less branching than those serving the open eye. Also, we found no difference in the morphology of the closed-eye axons between dark reared animals and those raised under binocular conditions. Thus, dark rearing did not restore the LGN axons that had been retracted by MD at a young age. However, both dark reared animals and those raised under binocular conditions significantly recovered from the shrinkage of the soma size of LGN neurons. These results are consistent with the previous report showing that simply reopening the closed eye restores the soma size of LGN neurons in cats (O'Leary et al., 2012). The soma size of LGN neurons is restored even when MD is immediately followed by dark rearing or binocular eyelid suture (Duffy et al., 2015). Therefore, the recovery of LGN soma size occurs when the inputs from the two eyes are balanced and does not require visual experience. As for the recovery of cortical responses, visual inputs to the closed eye is important since the visual acuity of the amblyopic eye gradually improves under binocular conditions after deprivation with or without dark rearing (Mitchell, 1988; Duffy and Mitchell, 2013). Taken together, these results indicate that the three major effects of MD on individual neurons, eye preference of cortical neurons, and soma size and axonal morphology of LGN neurons, have distinct mechanisms and require different restoration approaches.

In the present experiments, functional recovery of visual cortical responses to the closed eye after dark rearing occurred in the absence of restoration of afferent axons. Dissociation between measures at different levels, such as morphology, physiology, and behavior, have been reported previously. After reverse monocular deprivation in cats for 10 days at P40, cortical responses to the originally closed eye restored and became dominant over the originally open eye. However, at this time, the geniculocortical axons of both eyes had retracted compared to those of the normal animals (Antonini et al., 1998). When reverse deprivation at 5 weeks of age was followed by binocular recovery for several weeks, cortical OD distribution resembled to that of the normal animals but visual acuity remained very low in both eyes (Murphy and Mitchell, 1986). The recovery of cortical responses in the present study might be caused by a change in intracortical circuits rather than in input axons from the LGN. Dark rearing followed by reverse deprivation increases dendritic spine density in all layers of the visual cortex of rats (Montey and Quinlan, 2011). This study also showed that reverse deprivation after dark rearing increases the amplitude of the thalamocortical visually evoked potential. Therefore, in the present experiments, the efficacy or number of synaptic connections between cortical neurons and LGN axons serving the closed eye might increase, leaving the major structure of input axons unchanged. Even in that case, the diminished axonal branches may affect the recovery of visual function by limiting the scaffold for synapse formation transmitting the information from the closed eye. Therefore, dark rearing can enhance the recovery of visual acuity of and cortical responses to the closed eye, but it has limited or no effect on depth perception (Mitchell et al., 2016) and axonal morphology as demonstrated in the present study.

## Data Availability Statement

The original contributions presented in the study are included in the article, further inquiries can be directed to the corresponding author.

## Ethics Statement

The animal study was reviewed and approved by the animal care committee of the Tottori University.

## Author Contributions

TG and YH designed the experiments and wrote the manuscript. TG, KK, KO, TA, and YH performed physiological experiments. TG, AK, KT, CY, HO, AM, and YH performed anatomical experiments. All authors contributed to the article and approved the submitted version.

## Conflict of Interest

The authors declare that the research was conducted in the absence of any commercial or financial relationships that could be construed as a potential conflict of interest.
