# Supplementary material for: Dark Rearing Promotes the Recovery of Visual Cortical Responses but Not the Morphology of Geniculocortical Axons in Amblyopic Cat
Source: Front Neural Circuits. 2021 Apr 16;15:637638. doi: 10.3389/fncir.2021.637638 (PMC8085520; doi:10.3389/fncir.2021.637638)
Supplement: Supplementary file 1 [file Presentation_1.pdf]

Supplementary data

Dark Rearing Promotes the Recovery of Visual Cortical Responses but not the Morphology of Geniculocortical Axons in Amblyopic Cat

Takahiro Gotou, Katsuro Kameyama, Ayane Kobayashi, Kayoko Okamura, Takahiko Ando, Keiko Terata, Chihiro Yamada, Hiroyuki Ohta, Ayaka Morizane and Yoshio Hata

Supplementary figures 1 and 2

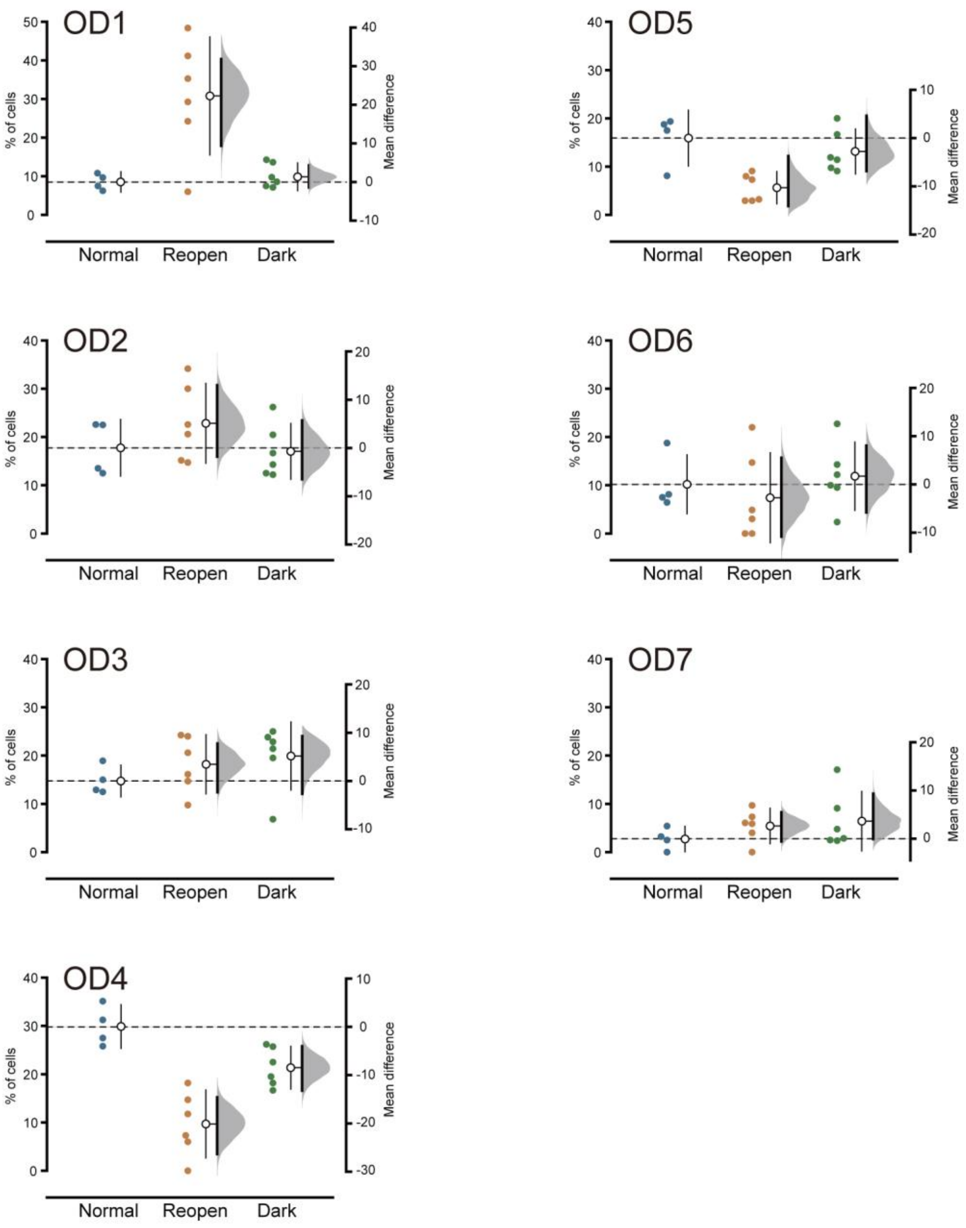

Supplementary Figure 1.

Comparison of ocular dominance in Reopen and Dark groups with Normal group. The data is a breakdown of Figure 1B. The proportion of cells with each ocular dominance score in individual animals is plotted as a filled circle on the left axes. The open circles with thin vertical bars indicate the mean and SD of each group. The right axes indicate the mean difference. The thick vertical bars represent the 95% confidence interval of mean difference with a bootstrap sampling distribution. In Reopen and Dark groups, OD1 and 7 represent cells which respond to the open- and closed eyes, respectively.

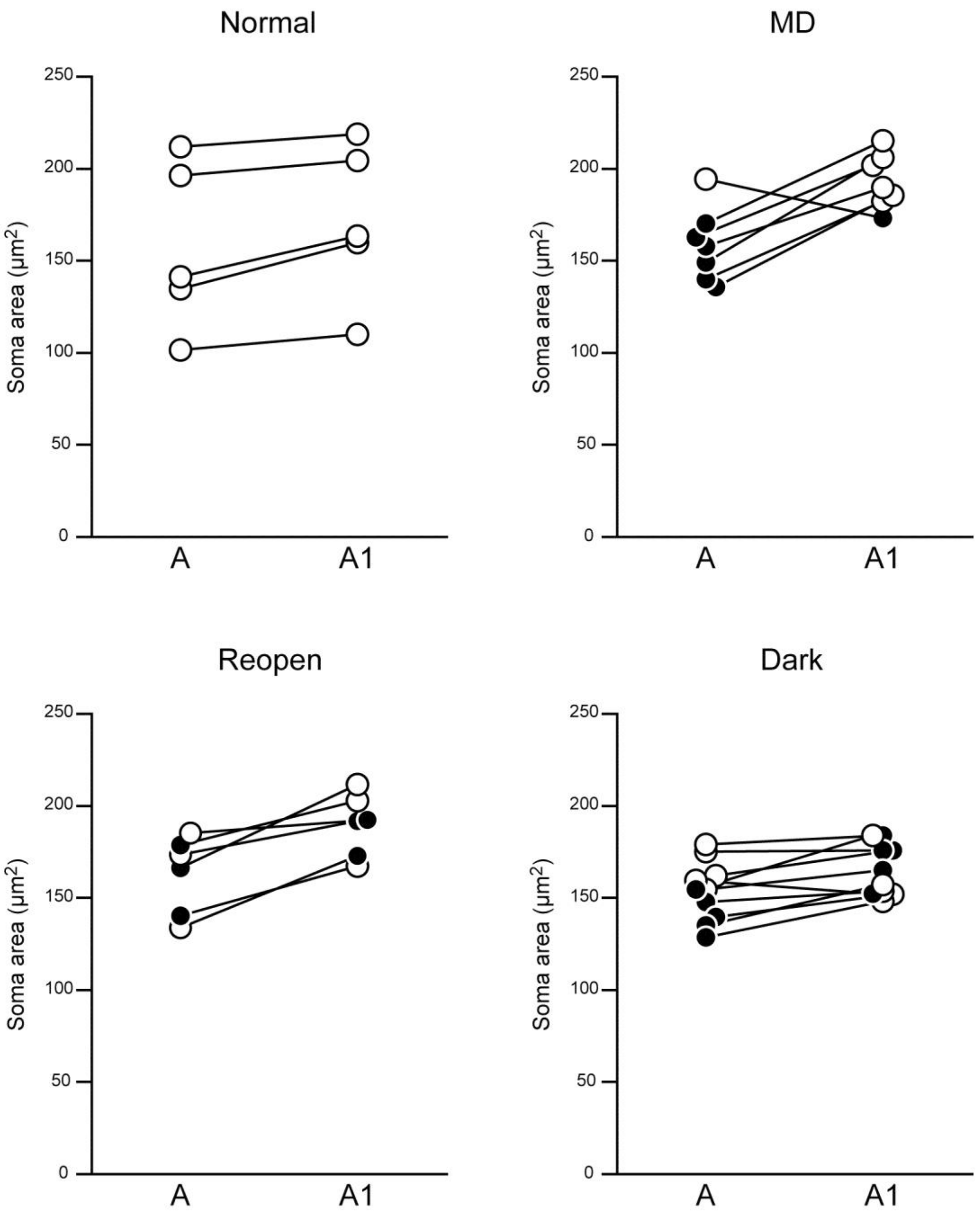

Supplementary Figure 2.

Soma area of LGN neurons in individual animals. The mean soma area of layer A and A1 of the same animal is connected by a line. The open and closed symbols represent the open-eye and closed-eye recipient layer, respectively.
